# A Novel 3D Bioprinter Using Direct-Volumetric Drop-On-Demand Technology for Fabricating Micro-Tissues and Drug-Delivery

**DOI:** 10.3390/ijms21103482

**Published:** 2020-05-14

**Authors:** Brian E. Grottkau, Zhixin Hui, Yonggang Pang

**Affiliations:** The Laboratory for Therapeutic 3D Bioprinting, Department of Orthopaedic Surgery, Massachusetts General Hospital, Harvard Medical School, Boston, MA 02114, USA; zhui@mgh.harvard.edu

**Keywords:** direct-volumetric, drop-on-demand, 3D bioprinter, 3D bioprinted tissues, drug-delivery model

## Abstract

Drop-on-demand (DOD) 3D bioprinting technologies currently hold the greatest promise for generating functional tissues for clinical use and for drug development. However, existing DOD 3D bioprinting technologies have three main limitations: (1) droplet volume inconsistency; (2) the ability to print only bioinks with low cell concentrations and low viscosity; and (3) problems with cell viability when dispensed under high pressure. We report our success developing a novel direct-volumetric DOD (DVDOD) 3D bioprinting technology that overcomes each of these limitations. DVDOD can produce droplets of bioink from <10 nL in volume using a direct-volumetric mechanism with <± 5% volumetric percent accuracy in an accurate spatially controlled manner. DVDOD has the capability of dispensing bioinks with high concentrations of cells and/or high viscosity biomaterials in either low- or high-throughput modes. The cells are subjected to a low pressure during the bioprinting process for a very short period of time that does not negatively impact cell viability. We demonstrated the functions of the bioprinter in two distinct manners: (1) by using a high-throughput drug-delivery model; and (2) by bioprinting micro-tissues using a variety of different cell types, including functional micro-tissues of bone, cancer, and induced pluripotent stem cells. Our DVDOD technology demonstrates a promising platform for generating many types of tissues and drug-delivery models.

## 1. Introduction

3D bioprinting technology deposits bioink, including live cells [1], biomolecules [2], and scaffold material [3], in a spatially controlled manner. It can be divided into two categories based on dispensing technologies: extrusion-based and droplet-based. The former deposits cells and biomaterials to a substrate by direct-contact [4]. The latter, especially drop-on-demand (DOD) technologies, are superior for generating micro-tissues, which are sub-millimeter constructs engineered to mimic the structures and functions of native tissues [5,6,7]. Inkjet, laser-based, and valve-based technologies are currently used to achieve DOD [7]. Inkjet technology is limited to ultra-low viscosity bioink and the droplets dispensed by both inkjet and laser tend to dry quickly [8]. Because of these limitations, valve-based bioprinting is most frequently used. Unfortunately, it also has several drawbacks: (1) It uses an indirect volumetric mechanism relying on pressure, valve-open time, and viscosity of the bioink. When any of these parameters change, the volume will also change. Even after extensive calibration and dispensing, volume inconsistency still exists because cell-sedimentation alters the viscosity of the bioink [8]. (2) Cells are pressurized during the entire course of bioprinting, which negatively impacts cell viability [9]. (3) Current DOD technologies can only accommodate relatively low viscosity bioinks, which have been reported to generate a less stable 3D structure [10]. (4) Cell concentrations > 3 × 10^6^ /mL result in severe nozzle clogging so concentrations < 1 × 10^6^ /mL are used [11]. This limits DOD’s capability in recapitulating tissues that are composed of cells at high concentrations. 

To overcome these problems, we developed a novel direct-volumetric DOD (DVDOD) technology for 3D bioprinting. It has two special features: (1) it drives out the bioink using a linear direct-drive mechanism so that the dispensed bioink is controlled in a direct-volumetric manner, (2) it uses pulsed air to release the bioink from the nozzle. The bioprinter can precisely dispense droplets of bioink as low as 10 nL with < ± 5% volumetric percent accuracy. The pressure exerted on the cells ranged from 5 to 60-fold less than that in the valve-based technology. The pressure occurs during bioink driving-out and drops to zero when it is not activated. This further protects the cells from pressure-related cell death. Our bioprinter also dispenses bioinks with viscosities up to 28-fold greater than the valve-based technology can accommodate. We have successfully generated functional tissues using alginate hydrogel and several different cell types with our novel DVDOD 3D bioprinter. 

## 2. Results

### 2.1. Volume Accuracy Measurement

**Figure 1:**
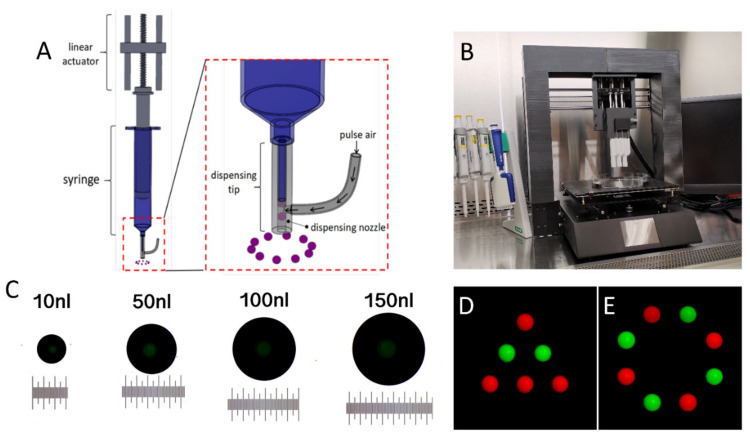
(**A**) Illustration of the dispensing mechanism of the DVDOD 3D bioprinting technology. (**B**) A representative set-up of a DVDOD 3D bioprinter. (**C**) Representative images of food dye solution dispensed into mineral oil at specific input volumes. The minimal scale equals 10 µm. (**D**,**E**) Representative patterned droplets dispensed onto the surface of a Petri dish. Green and red droplets were dispensed from two discrete dispensing units.

Figure 1C shows representative images of the bioprinted droplets of 10 nL, 50 nL, 100 nL, and 150 nL using DVDOD. The dispensing volume accuracies are between ± 3.15% and ± 4.83% using the input values between 10 nL and 350 nL.

### 2.2. Volumetric Patterning 

Accurate patterns of droplets were accomplished with multiple print units by dispensing droplets of fluorescein isothiocyanate–dextran (FITC-dextran) (green) and Texas-Red-dextran (red) (Figure 1D,E). All the droplets show a consistent center-to-center distance. No satellite droplet was observed, and the circularity of any individual droplet was >99%.

### 2.3. Bioprinting for High-Throughput Assays

**Figure 2:**
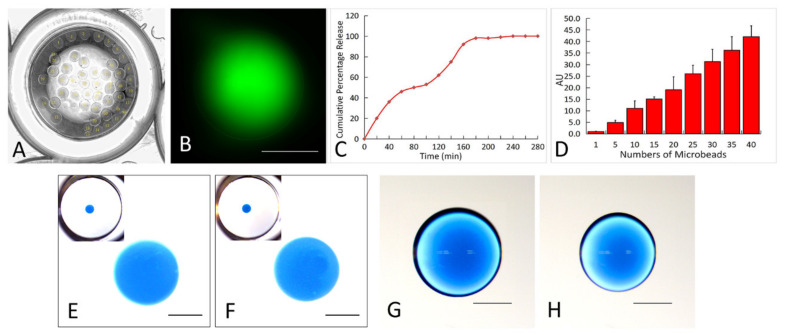
(**A**) Demonstration of the high-throughput assay. A representative phase contrast image of 40 alginate beads within a well of a 96-well plate. The beads were directly dispensed and formed within the well. (**B**) A representative fluorescent image of FITC-dextran releasing from an alginate bead within a well of a 96-well plate. (**C**) The accumulative percentage release curve of the FITC-dextran from the alginate beads. (**D**) The dose-release curve of the FITC-dextran incorporated alginate beads. All data were normalized to the amount of FITC-dextran released from a single bead. (**E**–**H**) Representative images of droplets of blue dextran-incorporated alginate, with high (F and H) and low (**E**,**G**) viscosities, which were dispensed into the CaCl_2_ solution (**E**,**F**, the images at the upper left corners are the global view of an entire well in a 96-well plate and the images in each center show the zoomed-in views) and Petri dish surface (**G**,**H**). The droplet with high viscosity (**H**) demonstrates smaller diameter than the low viscosity one (**G**) on the Petri dish surface. Each scale bar is 500 µm.

With the FITC-dextran as a model drug, we tested the feasibility of direct bioprinting drug-loaded beads into the wells of a 96-well plate. The specific number of alginate beads (up to 40, Figure 2A) were successfully generated by directly bioprinting into each well of a 96-well plate. The FITC-dextran released from the alginate beads into the phosphate-buffered saline (PBS) within a well is shown in Figure 2B. The percentage releases over time (Figure 2C) and among different numbers of beads (2D) were analyzed. 

### 2.4. High Viscosity Bioprinting

As shown in Figure 2F, using high viscosity alginate hydrogel, we successfully generated spherical beads in the CaCl_2_ solution while there was no bead distortion or satellite droplets observed. When this is compared with bioprinting the same volume of low viscosity alginate hydrogel (Figure 2E), there were no significant differences in terms of the circularity and diameter. When alginate droplets were dispensed onto the Petri dish surface, highly circular (in XY plane) droplets were generated and the circularities show no significant difference between the two groups (Figure 2G,H). However, a droplet of high viscosity alginate hydrogel (Figure 2H) has a smaller diameter. 

### 2.5. Pressure Value and Cell Viability

**Figure 3:**
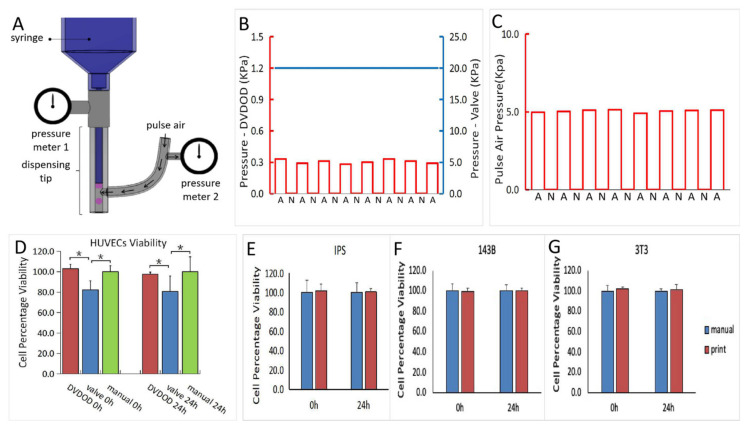
(**A**) Illustration of the pressure measurement set-up in the DVDOD. (**B**) The plots of pressure measured from the syringes of the DVDOD (red, A: syringe-moving is activated, N: syringe-moving is not activated) and valve-based printing technology (blue). (**C**) The plot of the pressure measured from the pulsed air in the DVDOD. (**D**) Cell percentage viability of HUVECs in the DVDOD, valve-based technology, and the manual operation at 0 h and 24 h. All the values were normalized to those of manual operation. * represents significant difference (*p* < 0.05*)*. (**E**–**G**) The cell percentage viabilities of Induced Pluripotent Stem (IPS), 143B, and MC3T3-E1 cells at 0 h and 24 h after bioprinting using DVDOD. All the values were normalized to those of manual operation. There was no significant difference observed in any cell type between the DVDOD and manual operation.

As shown in the pressure curves in Figure 3B,C, when bioprinting human umbilical vein endothelial cells (HUVEC) cells at a concentration of 1 × 10^5^ /mL, only about 0.3 kPa and 5 kPa are required to drive the fluid out of the syringe tip and nozzle respectively. Both pressures are applied in non-continuous mode so that cells are only subject to the pressure during the fluid-driving process (1 millisecond or less). The cells are subject to no measurable pressure in the syringe while the plunger is not activated. Conversely, in the valve-based bioprinting process, the cells are subject to pressure throughout the entire time course of printing and require higher pressure for dispensing. 

HUVECs from the same source were loaded into our DVDOD bioprinter and into a pneumatic syringe, which is normally used in a valve-based bioprinter. The HUVECs demonstrated no viability alteration at 0 h and 24 h in the DVDOD bioprinter system but did show significant 15% (0 h) and 17% (24 h) viability decreases (*p* < 0.05) in the pneumatic syringe system (Figure 3D). None of the additional 3 cell types, including MC3T3-E1, 143B and Induced Pluripotent Stem (IPS) cells, showed any viability alteration compared with manual pipetting as control at 0 h and 24 h after the processing in the DVDOD bioprinter system (Figure 3E–G).

### 2.6. 3D Bioprinting Live Cells with Alginate Hydrogel

**Figure 4:**
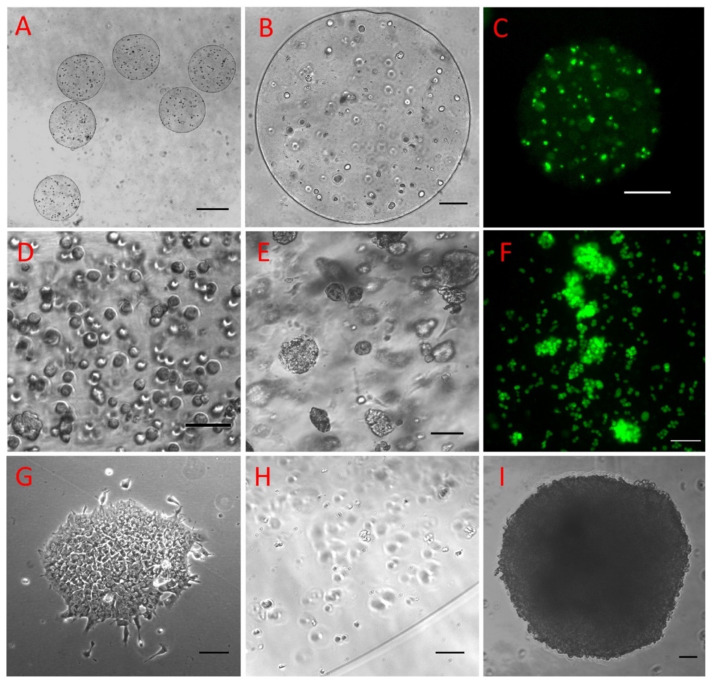
Typical views of 143B human osteosarcoma cells at a cell concentration of 1 × 10^6^ /mL immediately after the alginate hydrogel has solidified (**A**–**C**). The uniformity of the cell-incorporated alginate microbeads (**A**) and the cell distribution within a microbead (**B**) are shown. Cells were highly viable as demonstrated by the Calcein AM staining (**C**). The 143B cells were bioprinted at a concentration of 6 × 10^6^ /mL and were evenly distributed immediately after bioprinting (**D**). The 143B cells grew into nodules after 7 days of culture (**E**), and the nodules and cancer cells were highly viable cells after isolation from the microtissue (**F**). IPS cells grew into a clone in 2D culture (**G**) and they were evenly distributed at low concentration inside the alginate microbeads immediately after bioprinting (**H**) and grew into an embryonic body following in vitro culture (**I**). Bars: A: 100 µm, B: 500 µm, C: 200 µm, D,E: 200 µm, F–I: 100 µm.

Multiple types of lives cells, including MC3T3-E1 (Figure 4A–C), 143B (Figure 4D–F), and IPS cells (Figure 4G–I), were successfully bioprinted into tissues with alginate hydrogel. As shown in Figure 4C, Calcein AM staining demonstrated that the cells were viable. The bioprinted tissues also demonstrated their normal functions as demonstrated by the formation of cancer nodules inside the osteosarcoma tissue (Figure 4D–F) and the formation of the embryonic body in the IPS tissues (Figure 4G–I). 

## 3. Discussion

There are two approaches to engineer artificial tissues or organs. One is the top-down approach by seeding cells into a macro-size scaffold [12]. The other is a bottom-up approach in which larger tissues or organs are built from smaller building units, such as micro-tissues [5,13]. The latter has several advantages including the relative ease of cultivating the tissue into a more mature condition in vitro. This is because the small size makes it much easier to provide nutritional support to the entire tissue [14]. An additional advantage is that the tissues can be implanted via a minimally invasive approach, which generally results in lower morbidity in clinical applications [15]. 

Compared with conventional tissue engineering approaches, 3D bioprinting can precisely recapitulate tissues or organs with complex structures such as perfusable vascular networks [16,17], complex anatomical structures [17,18], complex environments [2], and structures composed of multiple materials [19]. The bottom-up bioprinting pathway requires bioprinted micro-tissues. Because DOD bioprinting technology has a higher resolution than extrusion-based bioprinting technology and can create small features with more precise control, it is preferred for generating micro-tissues. As mentioned previously, of the three current DOD technologies, the valve bioprinting is superior to the other two. However, it still has several major limitations. In the current project, we overcame these limitations by developing the DVDOD tissue bioprinter which provides a better solution for the creation of micro-tissues. We demonstrated its capabilities for high volumetric accuracy, viscous bioink printing, and cell viability protection. We also successfully bioprinted multiple functional tissues. 

Our DVDOD bioprinter employs a novel volumetric bioink-driving technique using linear actuator-driven syringes with a dispensing mechanism that is a specifically designed non-contact pulsed-air dispensing nozzle. We also tested the accuracy of volumetric dispensing. DVDOD cannot only dispense nanoliter-accurate individual droplets with < ± 5% volumetric percent accuracy, but it can also perform coordinated dispensing with multiple dispensing units to accurately pattern droplets. 

One major difference in the dispensing mechanism between the DVDOD technology and other DOD technologies, such as inkjet and value technologies, is how the force is applied to the bioink for dispensing. In our DVDOD technology, the force (pulsed air generated pressure) is only transiently applied to the bioink as it is being driven out of the dispensing tip. The volume of the dispensed droplet is in the nanoliter range. In all other technologies, the dispensing force is applied to all the bioink throughout the entire bioprinting process. The DVDOD technology requires a much lower level of force to release the bioink from the nozzle than the valve technology. Our unique dispensing mechanism allows the DVDOD bioprinter to print bioinks with high viscosity (viscous materials and high concentrations of cells) and preserves the cell viability during the printing process. 

Valve bioprinting technology can only print bioinks with viscosities < 70 cP. This limitation is due to its dispensing mechanism: opening the valve to let the pressurized bioink travel through and cutting off the stream of bioink by closing the valve. Once the viscosity is above 70 cP, non-circular and satellite droplets of bioink form and stick around the outside nozzle without dropping to the substrate. This constraint compromises its performance and limits its application. Higher viscosity bioink usually generates stronger structures thus increasing printability and resolution. Some biomaterials with viscosities that are above 70 cP cannot be printed with valve technology. Using our DVDOD technology, we were able to successfully dispense bioink with a viscosity above 2000 cP and a cell concentration of 6 × 10^6^ /mL, which are more than 28 times and 6 times higher than the maximum viscosity and maximal concentration that the valve technology can handle, respectively. 

Biomaterial carriers are widely used for therapeutic drug deliveries [20,21] and the emerging bioprinting technologies have the unique advantages of generating drug-delivery vehicles precisely [22]. Our DVDOD technology has the capability of accurately delivering droplets to a specific location in a volumetric manner and we also demonstrated its capability in a high-throughput assay. As a proof-of-concept, we chose a drug-delivery model using FITC-dextran as a model drug. The 3D printed drug-incorporated hydrogel bead demonstrated a typical drug release profile proving that we can control the amount of the released drug over time. In addition, we were able to control the amount of drug delivered at a specific time by changing the number of beads. Even though the high-throughput assay was modeled in a drug-controlled release manner, it could also be modeled using cell-incorporated hydrogel. We plan to study this systematically in the future. 

Another drawback with the other DOD technologies is that the bioink is pressurized throughout the entire process of printing which negatively affects cell viability [23]. In this current study, we analyzed the effect of pressure on cells using HUVECs-a widely used cell type in bioprinting. We observed that a pressure above 10 kPa reduced the cell viability to averages of 85% and 80% relatively to the non-pressured control at 0 h and 24 h, respectively. In contrast, the DVDOD requires a much lower level of pressure to drive the bioink. The pressure inside the syringe drops to zero while the plunger is static. The pressures inside the syringe and from the pulsed air are applied for a millisecond or sub-millisecond during one cycle of bioprinting. As a result, the DVDOD 3D bioprinter demonstrated no decrease in cell viability after printing.

After characterizing our DVDOD bioprinter, we bioprinted 3 types of cells representing 3 types of applications for micro-tissues: (1) bioprinting MC3T3-E1 cells into bone tissues, (2) bioprinting 143B cancer cells into cancer tissues and (3) bioprinting IPS cells into IPS tissues. The anticipated applications of the tissues are: (1) bone tissues for treating orthopedic conditions delivering micro bone tissue to the bone defect site in a minimally invasive manner, (2) cancer tissues for personalized cancer therapy and drug discovery by screening sensitive drugs for a specific patient and finding candidate drugs from a compound library, and (3) IPS tissues which provides a large amount of nearly identical tissue replicates as a developmental biology model. 

As a proof-of-concept study, we did not report variety types of biomaterials in the current project. However, we have tested hydrogels other than alginate that can be applied using our DVDOD technology. Considering the working mechanism of our DVDOD technology, we anticipate that it will be applicable to many hydrogels that have been reported previously using other DOD or extrusion technologies. 

In conclusion, we have developed a novel volumetric 3D bioprinting system with nanoliter level volume accuracy. It functions in a DOD manner with precise spatial deposition capability in a low or high-throughput mode. It can dispense bioinks comprised of high concentrations of cells and/or high viscosity biomaterials. As a proof-of-concept, we also developed a high-throughput drug-delivery model and generated functional tissues using precursor-osteoblasts, cancer cells, and IPS cells. Our DVDOD technology holds promise in generating many types of micro-tissues and drug-delivery models. 

## 4. Materials and Methods 

### 4.1. DVDOD 3D Bioprinter Design and Operation

The design of the DVDOD 3D bioprinter was documented in detail previously [24]. It is constructed of multiple dispensing units mounted on a three-axis linear motion system (Figure 1A,B). Each axis of the linear motion unit controls the position of the dispensing unit in the X, Y, and Z coordinates respectively and they collectively control the position of the dispensing unit precisely. Each dispensing unit includes a syringe controlled by a linear actuator. The volume of bioink driven out of the dispensing tip is in linear proportion to the linear actuator motion. The linear actuator drives the bioink into the nozzle. There is an air path connected to the nozzle. The pulsed air flows through the air path and pushes the bioink, which can include cells, hydrogels and biomolecules, from the nozzle out the dispensing tip orifice. Therefore, by moving the plunger a specific distance and driving the pre-determined volume of bioink out of the nozzle with pulsed air, the bioprinter can generate direct-volumetric droplets of bioink. Multiple coordinated dispensing units control the position of the dispensed droplets. The system also includes units that control temperature and humidity. The bioprinter is placed in a biosafety cabinet (Figure 1B) and the manipulations are carried out under sterile conditions. 

The DVDOD bioprinter can work in one of the following workflows according to the purposes required: (1) Bioprinting 3D micro-tissues: different cells and different hydrogels are loaded into the dispensing units. Cells and hydrogel(s) are dispensed to the substrate in a droplet over droplet manner. The 3D architecture is controlled by controlling the volume and the dispensing location of the droplets. In some applications, the cells and hydrogel(s) can also be dispensed into a solution in a volume and location-controlled manner. (2) Bioprinting for drug delivery: Drug-incorporated hydrogel(s) is (are) dispensed over a substrate or into a solution for polymerization. The location control is also important for dispensing into a solution because it prevents the collision and merging of droplets in the solution (see 4.3 for the detail of the application). 

### 4.2. Bioprinting Volume Accuracy Evaluation

Because single low volume (10 nL–1 nL) droplets evaporate quickly on a hard surface, we dispensed bioink composed of distilled water and green food dye into mineral oil. Water droplets form nearly perfect spherical shapes in mineral oil and droplet volume can be measured accurately [25]. The images of the droplets were captured and their volumes were analyzed through Image J (National Institutes of Health, Bethesda, Maryland, USA) [26]. Percent accuracy is expressed as a percent difference and was calculated using the following equation:

% difference = |” measured value” − “input value” | / “input value” × 100%

### 4.3. Droplet Patterning Coordinated by Multiple Printing Units

To demonstrate the spatial position capabilities of our DVDOD bioprinter, we used two dispensing units loaded with bioink composed of low viscosity (≤12 cP) alginate hydrogel (Sigma-Aldrich, St. Louis, MO, USA) mixed with FITC and Texas Red labeled dextran (Invitrogen, Carlsbad, CA, USA), respectively. Different patterns of droplets were used. To prevent liquid drying during the assay, 1 µL droplet was used to dispense onto the surface of a Petri dish. Images were captured using an epifluorescence microscope (Nikon, Minato City, Tokyo, Japan). 

### 4.4. Bioprinting for a High-Throughput Assay 

FITC-dextran was incorporated into the alginate beads as a model drug. A high-throughput drug control-release assay was performed by directly bioprinting and generating alginate beads in the wells of a 96-well plate. The alginate solution at 2% concentration was mixed with FITC-dextran and 300 µL of 102 nM calcium chloride (CaCl_2_) was added into the wells. The alginate droplets were directly dispensed into the wells using the following numbers of droplets per well: [1, 5, 10, 15, 20, 25, 30, 35, 40]. After solidification, the CaCl_2_ solution was replaced with a PBS solution. Aliquots of 100 µL PBS solution were sampled from each well immediately and every 20 min thereafter, replenishing each time. The fluorescence intensity of the released FITC-dextran was analyzed by a fluorescence plate reader (Perkin Elmer, Waltham, MA, USA). 

### 4.5. High Viscosity Bioink Printing

High viscosity alginate (rated viscosity > 2000 cP at 2%, Sigma-Aldrich, St. Louis MO, USA) was used for high viscosity bioink printing tests. It has a viscosity that is 28-fold and 66-fold higher than the upper limit capability of valve-based and inkjet bioprinting technologies, respectively. Alginate was dissolved in distilled water and mixed with blue dextran (Sigma-Aldrich, St. Louis, MO, USA), to make the final gel 2% *w*/*v* solution, and loaded into the dispensing unit. Droplets of 500 nL each were dispensed into a 102 nM CaCl_2_ solution and onto the surface of a Petri dish. The generated beads were imaged by stereomicroscopy. When using valve bioprinting with high viscosity bioinks, satellite droplets are generated that stick to the nozzle orifice [27] making the high viscosity bioink non-printable. Therefore, we analyzed whether satellite droplets were generated or any bioink residual sticks around the DVDOD nozzle. The circularities of the droplets were also analyzed. 

### 4.6. Cell Culture 

IPS cells (courtesy of Dr Hongmei Mou) were maintained on Geltrex coated plates (Life Technologies, Carlsbad, CA, USA) in feeder-free culture in mTesr1 medium (STEMCELL, Canada) at 37 °C with 5% CO_2_. At 70% confluency, cells were passaged with Accutase (STEMCELL, Vancouver, Canada). Using the protocols reported previously [18,28], HUVECs (Lonza, Basel, Switzerland) were grown in EGM-2 BulletKit (Lonza, Basel, Switzerland). 143B human osteosarcoma cells (ATCC) and preosteoblast MC3T3-E1 cells (ATCC) were cultured in Dulbecco’s Modified Eagle’s Medium (DMEM; Life Technology, Carlsbad, CA, USA) with 10% FBS. Cells were incubated under 37 °C with 5% CO_2_. The medium was changed 2-3 times per week. At 80% confluency, the cells were passaged using 0.05% trypsin-EDTA (Life Technology, Carlsbad, CA, USA). 

### 4.7. Pressure and Cell Viability Analysis

The pressures generated during the bioprinting process were recorded in the following three stages (Figure 3B,C):

1. Plunger-moving stage: plunger moves to drive out the bioink into the dispensing nozzle.
2. Bioink-loading stage: bioink is inside the dispensing nozzle while the plunger is static and pulsed air is not activated.
3. Bioink-dispensing stage: while plunger remains static, the pulsed air is activated, and the bioink is driven out of the dispensing nozzle.

HUVECs were adjusted to a concentration of 1 × 10^5^ /mL and baseline cell viability was analyzed using Calcein AM staining [18]. Using a typical pneumatic dispensing set-up, 1 mL of the HUVECs was pressurized for 15 min at 10 kPa (pressure used on HUVECs previously reported elsewhere [23]) onto the Petri dish through a 150 µm diameter nozzle. A cell suspension of 100 µL was aspirated for an immediate viability test and the remainder was cultured for an additional 24 h for another cell viability test. 

### 4.8. Live Cell Bioprinting 

Several different types of cells were bioprinted using alginate hydrogel including MC3T3-E1 osteoprecursor, IPS, and 143B cells. Sterile alginate hydrogel (2%) was mixed with the cells (concentrations up to 6 × 10^6^ / mL) and loaded into a dispensing unit. Three ml of CaCl_2_ solution was added to a 35 cm Petri dish and 500 nL /droplets were dispensed into the solution to form cell loaded beads. After 10 min, the beads were washed three times with PBS solution and cultured using the same medium used in 2D cell culture as documented above. 

## 5. Patent

Yonggang Pang, Brian Grottkau. Three-dimensional microtissue bioprinter. WO2017040975A1, PCT/US2016/050167. Assignee: The General Hospital Corporation.

## Abbreviations

DOD	drop-on-demand
DVDOD	direct-volumetric DOD
IPSs	induced pluripotent stem cells
HUVECs	human umbilical vein endothelial cells
CaCl_2_	calcium chloride
FITC	fluorescein isothiocyanate
PBS	phosphate-buffered saline
